# Polyploidy in haloarchaea: advantages for growth and survival

**DOI:** 10.3389/fmicb.2014.00274

**Published:** 2014-06-13

**Authors:** Karolin Zerulla, Jörg Soppa

**Affiliations:** Biocentre, Institute for Molecular Biosciences, Department of Biological Sciences, Goethe University FrankfurtFrankfurt, Germany

**Keywords:** *Haloferax volcanii*, archaea, polyploidy, gene conversion, desiccation, survival

## Abstract

The investigated haloarchaeal species, *Halobacterium salinarum, Haloferax mediterranei*, and *H. volcanii,* have all been shown to be polyploid. They contain several replicons that have independent copy number regulation, and most have a higher copy number during exponential growth phase than in stationary phase. The possible evolutionary advantages of polyploidy for haloarchaea, most of which have experimental support for at least one species, are discussed. These advantages include a low mutation rate and high resistance toward X-ray irradiation and desiccation, which depend on homologous recombination. For *H. volcanii,* it has been shown that gene conversion operates in the absence of selection, which leads to the equalization of genome copies. On the other hand, selective forces might lead to heterozygous cells, which have been verified in the laboratory. Additional advantages of polyploidy are survival over geological times in halite deposits as well as at extreme conditions on earth and at simulated Mars conditions. Recently, it was found that *H. volcanii* uses genomic DNA as genetic material and as a storage polymer for phosphate. In the absence of phosphate, *H. volcanii* dramatically decreases its genome copy number, thereby enabling cell multiplication, but diminishing the genetic advantages of polyploidy. Stable storage of phosphate is proposed as an alternative driving force for the emergence of DNA in early evolution. Several additional potential advantages of polyploidy are discussed that have not been addressed experimentally for haloarchaea. An outlook summarizes selected current trends and possible future developments.

## INTRODUCTION

Many species of eukaryotes are polyploid, and this is true for animals, plants, and lower unicellular eukaryotes. Last year, a special issue of Cytogenetic Genome Research focused on polyploidy and assembled many excellent reviews focusing on different aspects of polyploidy, including the mechanisms of its generation, the consequences for gene expression and genome biology, and the change of ploidy levels in both directions during evolution (e.g., Choleva and Janko, 2013; Wertheim et al., 2013; Hegarty et al., 2013; Weiss-Schneeweiss et al., 2013). Polyploidy is even observed in human tissues, e.g., in the liver and in tumors (Duncan, 2013; Mayfield-Jones et al., 2013). While there has been tremendous advances in recent years, it is still not clear whether polyploidy generally has a positive effect on the evolutionary success of a eukaryotic species (Madlung, 2013).

In stark contrast to eukaryotes, it has long been believed that prokaryotes (archaea and bacteria) are typically monoploid and contain a single copy of a circular chromosome, and this is still the current view of most reviews and textbooks (e.g., Madigan et al., 2012). Few exceptions were known and have been studied, e.g., the bacterium *Deinococcus radiodurans* that was isolated from irradiated meat and is highly resistant to X-ray irradiation and desiccation (Hansen, 1978). *D. radiodurans* has 5–8 copies of its chromosome and is thus “only” oligoploid (between 2 and 10 copies of the chromosome, more than 10 copies would be polyploid). Nevertheless, *D. radiodurans* can quickly and efficiently regenerate complete chromosomes from overlapping fragments of severely scattered chromosomes, which is a process that involves DNA synthesis and homologous recombination (Zahradka et al., 2006; Slade et al., 2009). This would not be possible for a monoploid species, and thus, survival in DNA damaging conditions is an obvious evolutionary advantage of the oligoploid of *D. radiodurans*.

In recent years, results have accumulated showing that *D. radiodurans* and a few additional, long-known examples are by no means seldom and exotic exceptions, but that many species of archaea and bacteria are oligo- or polyploid (e.g., Komaki and Ishikawa, 2000; Breuert et al., 2006; Mendell et al., 2008; Michelsen et al., 2010; Tobiason and Seifert, 2010; Griese et al., 2011; Hildenbrand et al., 2011; Pecoraro et al., 2011). Currently it seems that the opposite from the traditional view is correct, i.e., that monoploid species are a small minority among archaea and bacteria.

Several species of haloarchaea have also been shown to be polyploid in all growth phases and at various growth rates, e.g., *Halobacterium cutirubrum, Halobacterium salinarum*, *Haloferax volcanii* (review: Soppa, 2011), *H. mediterranei* (Zerulla and Soppa, unpublished results), and several new isolates (Zerulla and Soppa, unpublished results). Until now, no haloarchaeal species has been found to be monoploid; therefore, it might be that polyploidy is a general trait of haloarchaea. Thus, haloarchaea seem to be a suitable group of prokaryotes for studying polyploidy, its evolutionary advantages, and the molecular mechanisms of its regulation. Nine different possible evolutionary advantages of polyploidy for haloarchaea have recently been discussed (Soppa, 2013). For five of the advantages, experimental evidence had been published for haloarchaea at that time, and the remaining four were only theoretical considerations. In this contribution, we do not aim to reiterate the recent review, but to only shortly mention previously discussed angles and to focus on new results reported since this time, e.g., survival under extreme conditions on earth and perhaps elsewhere, the absence of an S-phase in the haloarchaeal cell cycle, the non-genetic role of genomic DNA as a phosphate storage polymer, and the possible driving force for the development of polyploidy in evolution.

## EVOLUTIONARY ADVANTAGES BASED ON HOMOLOGOUS RECOMBINATION

As mentioned above, it was shown long ago that the oligoploid bacterium *D. radiodurans* has a much higher resistance to DNA-damaging conditions than other bacteria with only one copy of their chromosome. Similarly, the polyploid haloarchaeon *H. salinarum* has been shown to be highly resistant to conditions that induce double strand breaks, i.e., X-ray irradiation and desiccation (Kottemann et al., 2005). Also, in this case, the chromosomes were scattered into many fragments, and complete chromosomes were re-generated by making use of overlapping fragments. In a further study, *H. salinarum* was challenged with increasing doses of X-ray irradiation, resulting in the selection of a mutant that had an even more enhanced irradiation resistance and was, in fact, believed to exhibit the highest resistance of all organisms on earth (DeVeaux et al., 2007). In the mutant, the expression of two genes encoding single strand DNA-binding proteins (Ssb) was highly up-regulated, indicating that the survival of DNA-damaging conditions involves homologous recombination in *H. salinarum*, like in *D. radiodurans*. In accordance with this view, overexpression of the homologous *rpaC* gene in *H. volcanii* resulted in an enhanced resistance against various DNA-damaging conditions (Skowyra and MacNeill, 2012). Based on these studies with haloarchaea, the role of the Ssb for the radiation resistance of *D. radiodurans* was recently investigated, and it was found that the *ssb* gene is not only essential, but furthermore, that the expression level is directly correlated with the degree of radiation resistance (Lockhart and DeVeaux, 2013).

It seems that polyploidy does not only confer resistance to DNA damaging agents but also results in a low rate of spontaneous mutations. The mutation rate of *H. volcanii* has been quantified in a genetic screen using the *pyrE* gene as a reporter, and it was found to be nearly one order of magnitude lower than that of other comparable mesophilic species (Mackwan et al., 2007). The authors proposed that the low mutation rate might be based on the polyploidy of *H. volcanii,* which enables the repair of mutated copies of the chromosome by making use of the information of wild-type copies that are simultaneously present in the cell.

The repair and the induction of mutations might be of evolutionary advantage. The presence of many copies of any given gene, termed gene redundancy, allows for the mutation of some of the copies without losing the wild-type information of the remaining copies. This generates heterozygous cells, which might be able to grow under unfavorable conditions that inhibit growth of the homozygous wild-type. Several reports about heterozygous cells in specific laboratory settings are available. For example, the selection of heterozygous *H. volcanii* cells that can grow in the absence of leucine and tryptophan, which is impossible for homozygous mutants, has been described (Lange et al., 2011). The presence of heterozygous cells under specific selection conditions has also been described for a methanogenic archaeon (Hildenbrand et al., 2011) as well as for several cyanobacteria (e.g., Spence et al., 2004; Takahama et al., 2004; Nodop et al., 2008). The ease of selection of heterozygous cells in polyploid species of diverse phylogenetic groups suggests that such cells can also arise in natural populations under appropriate selection conditions.

An alternative potential mechanism for the formation of heterozygous cells is the fusion of two cells with non-identical genomes. For *H. volcanii*, it has been shown that genetic transfer between different auxotrophic cells is possible and involves cytoplasmic bridges and most likely the fusion of cells (Mevarech and Werczberger, 1985; Rosenshine et al., 1989). For a population of *Halorubrum* growing in a saltern, it was revealed that the cells exchanged genetic information promiscuously and that the linkage equilibrium was extremely low and, in fact, approached that of a sexual population (Papke et al., 2004). This indicates that heterozygous cells also form in nature, at least for a while. Recently, it was shown that even cells of two different species could fuse (Naor et al., 2012). The resulting heterozygous cells were not stable, but cells with identical genomes emerged that had integrated between 310 and 530 kbp of the genome of the other species into the main genome. Natural populations of the genus *Halorubrum* in two salterns and one salt lake have been characterized, and it has been found that clusters can be formed but that barriers to genetic exchange between the different “species” is leaky, indicating that cell fusion between cells of different “species” might also occur in nature (Papke et al., 2007). This is most likely not confined to haloarchaea. Recently, it has been discussed that lateral transfer between different species of prokaryotes has occurred so massively that a tree-like reconstruction of phylogeny does not adequately describe what has happened in evolution, and a network-like reconstruction is more appropriate (Dagan, 2011; Dagan and Martin, 2009). Of course genetic exchange is not only possible via cell fusion but also through other mechanisms that require the intermediate formation of cells that are heterozygous at least for some of their genes.

## INTERMOLECULAR GENE CONVERSION IN THE ABSENCE OF SELECTION

**FIGURE 1:**
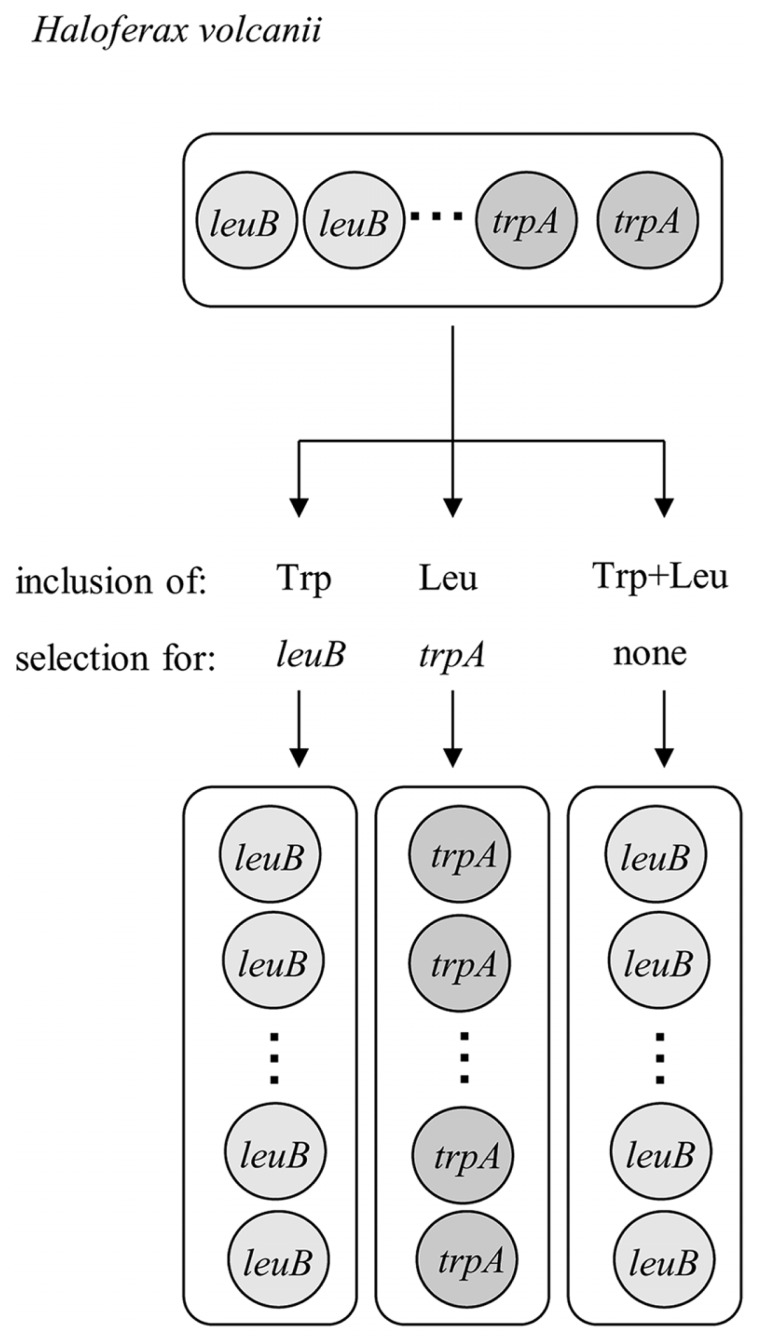
**Schematic overview of an experiment verifying that gene conversion operates in the absence of selection in *Haloferax volcanii* (changed version of Figure 1 in Soppa, 2013)**.

The repair of damaged or mutated copies of the chromosome using the wild-type information of other copies would require the un-reciprocal, intermolecular transfer of information from a donor to an acceptor molecule, a mechanism termed gene conversion. It has been shown that gene conversion indeed operates in *H. volcanii* and leads to the equalization of genome copies in the absence of selection (Lange et al., 2011). Shortly, a heterozygous strain was constructed that simultaneously contained two different types of chromosomes that had either the *leuB* or *trpA* gene at the *leuB* locus. Selection for the presence of either of the two amino acids led to an equalization of genomes in the direction of the respective essential gene, while the genomes lost the information of the other gene. Most importantly, gene conversion also led to an equalization of genome copies in the absence of any selection. In addition, gene conversion occurred in the predicted direction, which required a smaller amount of DNA synthesis than the other direction. The experiment is schematically shown in **Figure 1**. Equalization of genome copies in the absence of selection has also been shown to operate in methanogenic Archaea (Hildenbrand et al., 2011). These are the only two studies that concentrated on intermolecular gene conversion in prokaryotes, and one additional study verified the existence of intermolecular gene conversion in chloroplasts (Khakhlova and Bock, 2006). Many more studies are available that concentrate on intramolecular gene conversion, which results in the concerted evolution of gene families and is a mechanism of antigenic variation or phase variation (Santoyo and Romero, 2005; Palmer and Brayton, 2007). Even if intergenic gene conversion in polyploid prokaryotes is a neglected field of research, it can be predicted to occur in more bacterial and archaeal species than in *H. volcanii* and *Methanococcus maripaludis* and result in evolutionary advantages of polyploidy. In addition, it is an escape from “Muller’s ratchet” theory, which predicted that asexual polyploid species cannot exist because they would accumulate deleterious mutations (Muller, 1964; Lehman, 2003).

## LONG-TERM SURVIVAL AND SURVIVAL IN EXTREME ENVIRONMENTS

It has repeatedly been reported that haloarchaea have been isolated from ancient salt deposits that had remained undisturbed for geological times (e.g., Fendrihan et al., 2006; Park et al., 2009; Schubert et al., 2010; Gramain et al., 2011; Xiao et al., 2013). The salt deposits are in different parts of the world and have different ages, and the isolations were performed by several different research groups. Nevertheless, whether haloarchaea can survive for more than 100 000 years in salt deposits has been the matter of intense debate, and a counterargument has always been that the chemical stability of DNA is too small to allow its survival in an intact form over geological times (a good review of the different arguments is given by Grant et al., 1998). However, this counterargument does not hold true for polyploid species. As has been experimentally proven for *H. salinarum*, polyploid cells can regenerate intact chromosomes from scattered fragments (Kottemann et al., 2005). In liquid enclosures within halite crystals, it can be expected that the DNA damage will be small because irradiation and oxidative stress are absent. The maintenance energy for long-term survival, including the repair of double-strand breaks, might originate from lysis of some or even the majority of cells of the enclosed population. For *Escherichia coli,* it has indeed been shown that cell lysis promotes growth of the remaining population (Corchero et al., 2001). Recently, three haloarchaeal species have been freshly isolated from an ancient salt deposit, and their ploidy levels have been quantified. All three were shown to be polyploid, in agreement with the predication that polyploidy enables long-term survival (Jaakkola et al., submitted for publication).

It has recently been observed that haloarchaea rapidly change their morphology upon exposure to conditions of low water activity and form 3–4 small spheres from one rod-like cell (Fendrihan et al., 2012). These spheres could outgrow to normal rods in favorable conditions. It could be that diminishing the surface to volume ratio is part of the strategy for long-term survival. Furthermore, roundish particles had indeed been observed in fluid inclusions of old halite crystals (Schubert et al., 2010). Notably, such a strategy would be impossible for monoploid species because only one of the 3–4 spheres would obtain a copy of the chromosome. The presence of a potassium pump has been discussed as another aspect of the long-term survival strategy of haloarchaea (Kixmüller and Greie, 2012).

Haloarchaea have not only been claimed to survive geological times enclosed in ancient salt deposits but also been shown to survive at extreme places on earth, e.g., places with extremely low water activity (e.g., Wierzchos et al., 2012). It has been discussed that some of these places are somewhat reminiscent of the conditions on Mars, and it has been proposed that haloarchaea could possibly survive on Mars. Therefore, *Halococcus dombrowskii* was exposed to simulated Martian UV irradiation, and the survival rate was found to be high when the cells were in fluid inclusions in halite crystals (Fendrihan et al., 2009). Notably, a considerable fraction of *Halococcus sp.* cells survived two weeks in space (Mancinelli and Klovstad, 2000), indicating that even extraterrestrial travel, e.g., on meteorites, might be possible for haloarchaea. Even if life on Mars is currently a fantastic idea, it induces experiments showing under which extreme conditions haloarchaea can survive – on earth.

## RELAXED REPLICATION CONTROL

Typically the cell cycle is highly regulated, and cell cycle checkpoints guarantee that its progression is stopped when problems occur, e.g., when a septum is not formed and the cell does not divide before replication is completed. DNA synthesis is usually confined to the so-called S-phase of the cell cycle, which is situated between the G1 and the G2 phases. Synchronized cultures of *H. salinarum* have been used to study the progression of cell cycle events, e.g., cell cycle-specific cyclic transcript level changes (Baumann et al., 2007). Unexpectedly, pulse labeling of newly synthesized DNA with 5-bromo-2′-deoxyuridine revealed that *H. salinarum* does not have an S-phase, but that DNA synthesis is constitutive (compare Figure 6 in Zerulla et al., 2014a). This loss of temporal replication regulation is not accompanied by a general relaxation of cell cycle control, e.g., the inhibition of replication results in a total stop of cell division, although the cell has approximately 30 genome copies and could easily divide several times without DNA synthesis (Herrmann and Soppa, 2002). In addition, DNA segregation control is intact, and the two daughter cells obtain equal amounts of DNA (Breuert et al., 2006), in contrast to other polyploid species (see below).

## GENOMIC DNA AS A PHOSPHATE STORAGE POLYMER

**FIGURE 2:**
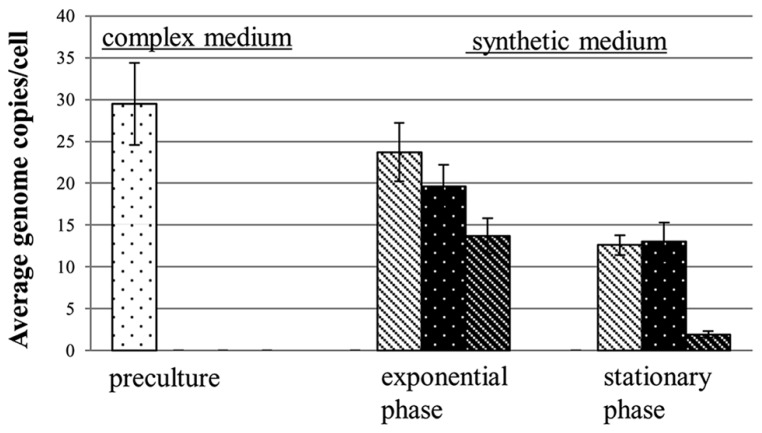
**Chromosome copy numbers of *H. volcanii* cells grown in synthetic media with two different phosphate concentrations and in the absence of external phosphate.** The copy numbers were quantified during mid-exponential growth phase and at stationary phase, as indicated (taken from Zerulla et al., 2014b).

Most of the genetic advantages of polyploidy discussed above require the presence of homologous recombination. Recently, an additional evolutionary advantage of polyploidy has been described that is independent of homologous recombination, namely the usage of genomic DNA as a storage polymer for phosphate (Zerulla et al., 2014b). The study initially aimed at clarifying whether *H. volcanii* can use external environmental genomic DNA as “food.” It could indeed be shown that *H. volcanii* can use external genomic DNA as a source for carbon, nitrogen, and phosphate (Chimileski et al., 2014; Zerulla et al., 2014a). However, the negative control lacking any external source of phosphate revealed that *H. volcanii* could grow to a limited extent under this condition and, thus, must have an intracellular phosphate storage pool. Quantification of the chromosomes showed that their copy number was dramatically decreased from approximately 30 to only 2 during growth in the absence of an external phosphate source (**Figure 2**). The cell number increased “only” 8.4-fold in the absence of phosphate, thus the decrease in chromosome copy number was higher than the increase in cell number. This indicates that *H. volcanii* used genomic DNA as a phosphate storage polymer in two different ways: (1) the cells could divide approximately three times in the absence of external phosphate, which is not possible for monoploid cells devoid of an alternative phosphate storage polymer such as polyphosphate, and (2) 1/3 of the genome copies were degraded to liberate phosphate for other phosphate-containing biomolecules lacking an intracellular storage pool, e.g., ATP, NADP^+^, phospholipids, phosphoproteins, and phosphosugars. The high copy number of *H. volcanii* implicates that one of the many biological functions of polyploidy is phosphate storage. The genetic advantages of polyploidy do not seem to require a copy number of 30, e.g., *D. radiodurans* has an extremely high resistance against irradiation and desiccation but harbors only eight copies of its chromosome. Re-addition of phosphate to phosphate-starved cells of *H. volcanii* led to fast and intense DNA synthesis and the copy number increased from 2 to 20 within 7 h.

**FIGURE 3:**
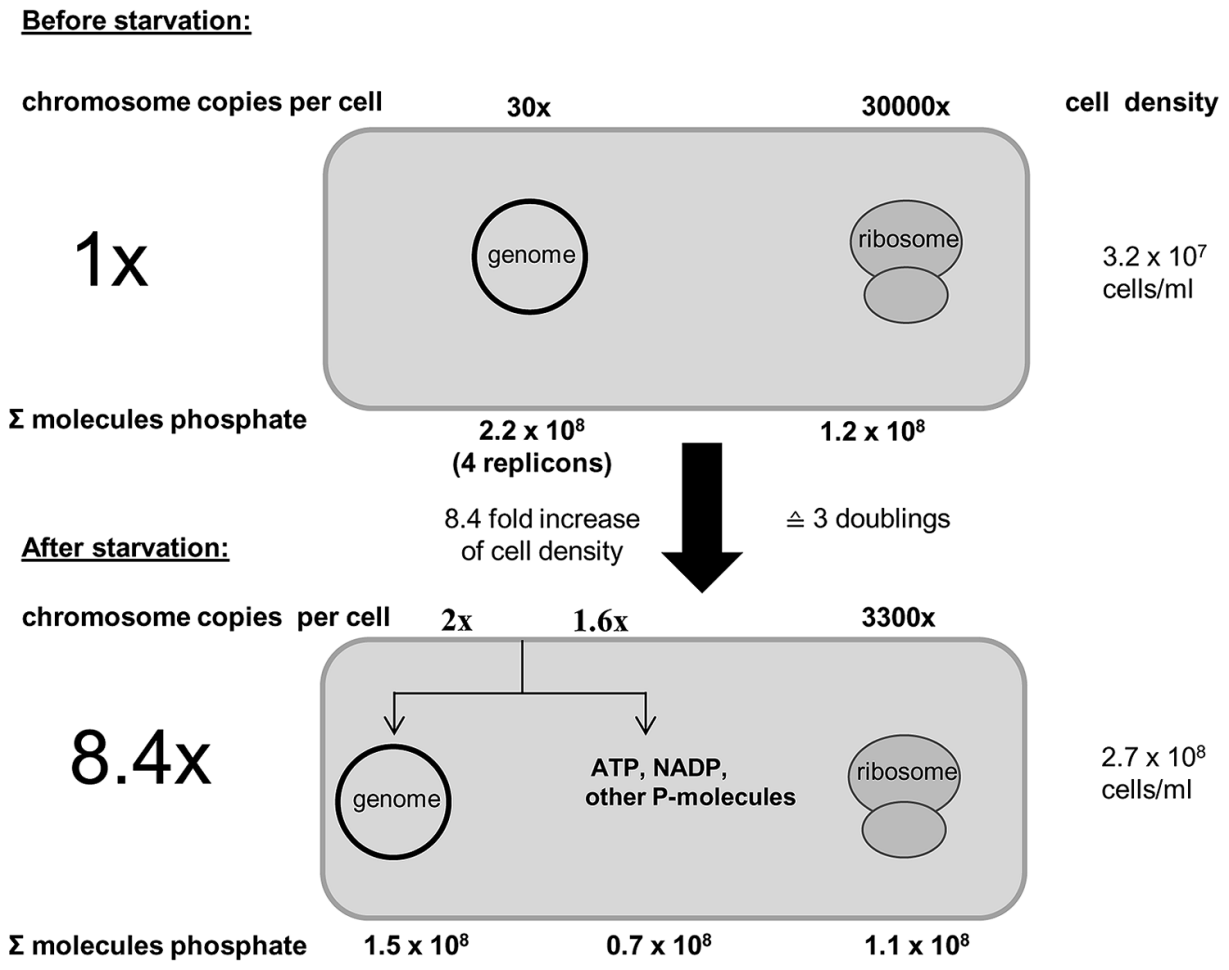
**Schematic overview of the phosphate balance of *H. volcanii* prior to and after growth in the absence of external phosphate.** It is taken into account that the cell number increased 8.4-fold during growth in the absence of phosphate. The number of chromosomes and ribosomes were quantified (taken from Zerulla et al., 2014b).

Further analyses revealed that genomic DNA seems to be the only intracellular phosphate storage polymer for *H. volcanii*. The number of ribosomes decreased in accordance with the increase in cell number, and thus, ribosomes were distributed to offspring cells, but not degraded to liberate phosphate, and no indication for the presence of polyphosphate could be found. **Figure 3** schematically summarizes the phosphate balance prior to and after growth of *H. volcanii* in the absence of phosphate.

**FIGURE 4:**
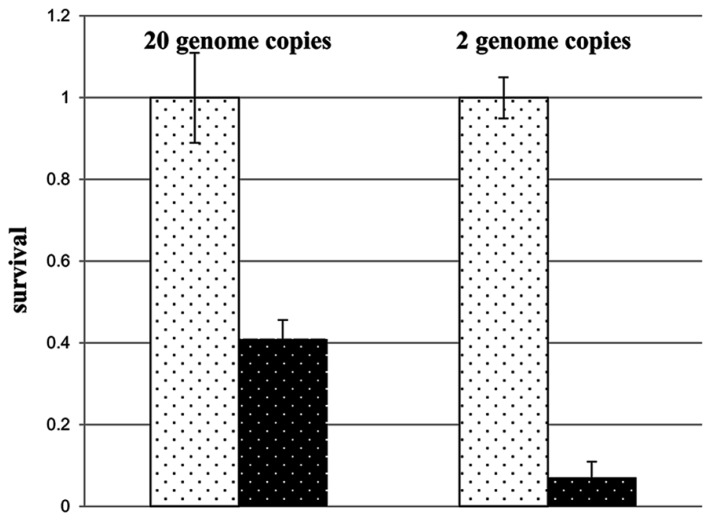
**Survival rates of *H. volcanii* cultures that entered a 12 day desiccation challenge with 20 chromosomes or with 2 chromosomes, as indicated (taken from Zerulla et al., 2014b)**.

In the first paragraphs of this contribution, a variety of genetic advantages of polyploidy were discussed, some or all of which might also apply to *H. volcanii*. The reduction of the chromosome copy number from 30 to 2 during growth in the absence of phosphate led to the prediction that concomitant genetic advantages should be diminished. This was indeed found to be the case. Comparison of the desiccation resistance of cells containing 20 and 2 copies of the chromosome revealed that the resistance of the former was fivefold higher than that of the latter (**Figure 4**). Therefore, if environmental conditions force *H. volcanii* to “choose” between several different advantages of polyploidy, genetic advantages are diminished in favor of cell multiplication. It will be interesting to reveal whether additional or all polyploid species also use this strategy, or whether other species abandon cell division in the absence of phosphate and retain the genetic advantages of polyploidy.

## THE POSSIBLE DRIVING FORCE FOR THE EMERGENCE OF DNA IN EVOLUTION

Several different and conflicting theories about the origin of life exist, including an autotrophic origin driven by natural gradients at geothermal vents and a heterotrophic origin in a “primordial soup” (Miller et al., 1997; Wächtershäuser, 1997; Lane et al., 2010; Neveu et al., 2013). However, all theories are in agreement that RNA predated DNA and that, at some point, free-living cellular life forms existed that had RNA as their genetic material. Much later, DNA was introduced into cellular life and replaced RNA as genetic material. The current view is that the selective driving force for the emergence of DNA was its higher stability in comparison with RNA, leading to a reduction in mutation rates and enhanced survival of cells with DNA-based genomes, which would rapidly outgrow cells with RNA-based genomes. Recently, an alternative explanation has been proposed that is based on the observation that *H. volcanii* uses DNA as a phosphate storage polymer in addition to its role as genetic material (Zerulla et al., 2014b). The alternative hypothesis is that, during the pre-DNA era, cells could grow and live with their RNA-based genomes, but, during phosphate limitation, growth was impossible in the absence of a phosphate-storage polymer. The driving force for the emergence of DNA would, in this view, have been its much higher stability compared to alternative phosphate storage polymers, e.g., polyphosphate, which is rather unstable. Therefore, the first “polyploid” cells would have had a high content of DNA without using it as genetic material. Only later would DNA have evolved its additional function as a genetic information storage polymer and then be selected for by its higher stability compared to RNA.

## POSSIBLE ADDITIONAL ADVANTAGES NOT EXPERIMENTALLY VERIFIED FOR HALOARCHAEA

Haloarchaea typically contain several large replicons, which are either regarded as several different chromosomes or as one chromosome and one or more mega-plasmids. It has been argued that *H. volcanii* contains four different chromosomes and one small plasmid because the former replicons all contain a replication origin composed of a repeated set of a sequence motif that is bordered by a gene encoding an Origin Recognition Protein (Hartman et al., 2010). The copy numbers of these replicons are not identical; therefore, the gene dosage of a given gene depends on its localization on a specific replicon, and the gene dosages can vary by more than a factor of four (Zerulla et al., 2014a). In addition, the copy numbers of the different replicons is typically growth phase-dependent, and the numbers are smaller in stationary phase than in exponential growth phase. Furthermore, growth phase-dependent copy numbers are independently regulated for the different replicons, and this has been shown for *H. volcanii* (Zerulla et al., 2014a), *H. mediterranei* (Liu et al., 2013), and *H. salinarum* (Breuert et al., 2006). The independent differential regulation of gene dosages in response to growth phase or varying environmental conditions opens the possibility that haloarchaea apply the regulation of replicon copy number for the global regulation of gene expression. However, until now, it was unclear whether gene expression in haloarchaea was correlated in a systematic manner with gene dosage. Only for one gene, the dihydrofolate reductase (*dhfr*) gene, is it clear that a higher gene dosage results in a higher expression level (Zusman et al., 1989). It is readily possible to select mutants of *H. volcanii* that are resistant to trimethoprim, a competitive inhibitor of DHFR. Many of these mutants carry amplifications of the genome region including the *dhfr* gene, and this was initially used to clone the gene.

Polyploid species might relax the strict control of DNA segregation and septum localization, without the danger of forming DNA-less daughter cells that waste the energy used for their production. It has in fact been shown that *Methanocaldococcus jannaschii* divides and generates daughter cells of different sizes with different DNA contents (Malandrin et al., 1999). However, relaxation of segregation control and cell division has not been reported for any haloarchaeal species.

A further attractive, yet untested, hypothesis is that the regulation of gene expression is different in monoploid and polyploid species. Monoploid prokaryotes contain just a single copy of a promoter of a typical gene. The copy number of transcription factors is also usually low. Therefore, regulation of gene expression depends on a stochastic sequence of on- and off-states of genes over time that is based on the off-rate and on-rate of the respective transcription factor at the respective promoter. This leads to differential protein compositions of cells of identical genotypes, and, indeed, single-cell analyses have revealed that populations of prokaryotes exhibit phenotypic variations (e.g., de Jong et al., 2012). In contrast, polyploid species similar to haloarchaea contain 20–30 copies of each promoter, and it is tempting to speculate that the copy numbers of transcription factors might be considerably higher than that in monoploid species. If this would be true, the regulation of gene expression in polyploid species would follow statistics rather than stochastics, and populations would be more uniform.

A further advantage that has not been verified for haloarchaea is the enlargement of cell size to the formation of “giant cells.” Giant cells escape predators that are specialized to feed on normal-sized prokaryotes. One example of a giant bacterium is *Epulopiscium,* which can reach a length of up to 600 μm. The cells are highly polyploid and have genome copy numbers of up to 10s of 1000s. It has been argued that cell enlargement depends on polyploidy because diffusion would be much too slow to distribute transcripts from a single genome throughout the volume of the cell (Mendell et al., 2008). Giant bacteria are found in several phylogenetic groups, and it has been described that all large bacterial species examined thus far are highly polyploid (Angert, 2012).

## OUTLOOK

It is now firmly established that several haloarchaeal species are polyploid and that polyploidy might even be a general trait of haloarchaea. However, genomes have been shown to be extremely flexible in evolution (Oliverio and Katz, 2014), and thus, many more haloarchaeal species must be tested before a generalization is possible. The molecular mechanisms of genome copy number regulation have not been unraveled. The major chromosome of *H. volcanii* contains four origins of replication, all of which can be deleted without loosing viability (Hawkins et al., 2013). This opens the possibility to construct small replicons as “haloarchaeal artificial chromosomes,” which carry a selected origin of replication that can be easily manipulated and used to study copy number regulation. In addition, it would be desirable to isolate isogenic strains with varying chromosome copy numbers, which would allow for systematically analyzing the advantages of polyploidy via the comparison of strains that are identical apart from their ploidy level. Furthermore, gene conversion has been primarily studied using eukaryotes ( >90% of the literature) and only poorly studied with bacteria, and fewer than five studies have been performed with archaea (Lawson et al., 2009). Moreover, intermolecular gene conversion has only been verified for two prokaryotic species. Thus, haloarchaea might help in the initial understanding of the importance and mechanism of intermolecular gene conversion in prokaryotes. In addition, haloarchaea might tremendously contribute to unraveling the importance of polyploidy for survival mechanisms over geological times, at very extreme environments on earth, or even on other planets.

## Conflict of Interest Statement

The authors declare that the research was conducted in the absence of any commercial or financial relationships that could be construed as a potential conflict of interest.
